# Temporal regulation of Lsp1 *O*-GlcNAcylation and phosphorylation during apoptosis of activated B cells

**DOI:** 10.1038/ncomms12526

**Published:** 2016-08-24

**Authors:** Jung-Lin Wu, Hsin-Yi Wu, Dong-Yan Tsai, Ming-Feng Chiang, Yi-Ju Chen, Shijay Gao, Chun-Cheng Lin, Chun-Hung Lin, Kay-Hooi Khoo, Yu-Ju Chen, Kuo-I. Lin

**Affiliations:** 1 Institute of Microbiology and Immunology, National Yang-Ming University, Taipei 112, Taiwan; 2 Genomics Research Center, Academia Sinica, Taipei 115, Taiwan; 3 Institute of Chemistry, Academia Sinica, Taipei 115, Taiwan; 4 Institute of Biochemistry and Molecular Biology, National Yang-Ming University, Taipei 112, Taiwan; 5 Institute of Biological Chemistry, Academia Sinica, Taipei 115, Taiwan; 6 Department of Chemistry, National Tsing Hua University, Hsinchu 300, Taiwan

## Abstract

Crosslinking of B-cell receptor (BCR) sets off an apoptosis programme, but the underlying pathways remain obscure. Here we decipher the molecular mechanisms bridging B-cell activation and apoptosis mediated by post-translational modification (PTM). We find that *O*-GlcNAcase inhibition enhances B-cell activation and apoptosis induced by BCR crosslinking. This proteome-scale analysis of the functional interplay between protein *O*-GlcNAcylation and phosphorylation in stimulated mouse primary B cells identifies 313 *O*-GlcNAcylation-dependent phosphosites on 224 phosphoproteins. Among these phosphoproteins, temporal regulation of the *O*-GlcNAcylation and phosphorylation of lymphocyte-specific protein-1 (Lsp1) is a key switch that triggers apoptosis in activated B cells. *O*-GlcNAcylation at S209 of Lsp1 is a prerequisite for the recruitment of its kinase, PKC-β1, to induce S243 phosphorylation, leading to ERK activation and downregulation of BCL-2 and BCL-xL. Thus, we demonstrate the critical PTM interplay of Lsp1 that transmits signals for initiating apoptosis after BCR ligation.

## Abstract (web summary)

B cell receptor (BCR) activation can trigger signalling causing apoptosis in order to eliminate auto-reactive B cells. Here the authors show that the O-GlcNAcylation and phosphorylation of lymphocyte-specific protein-1 are involved in a switch that regulates the initiation of apoptosis induced by BCR cross-linking.

## Graphical abstract

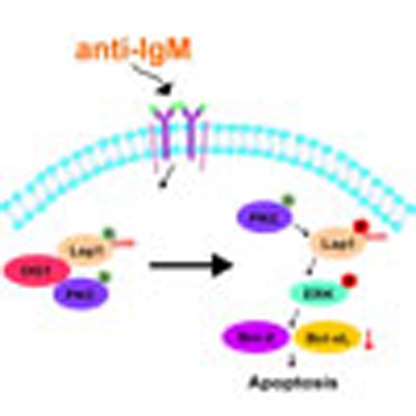

Bcell activation is critical for mounting antibody responses. Following antigen engagement with B-cell receptors (BCRs), a series of signalling cascades are triggered that involve the activation of spleen tyrosine kinase (Syk) and Lck/Yes-related Novel protein tyrosine kinase (Lyn), which phosphorylate phospholipase C gamma 2 (PLC-γ2) and Bruton's tyrosine kinase, respectively. PLC-γ2 and phosphoinositide-3-kinase (PI3K) further activate downstream pathways, including PLC-γ2/calcium/NFAT, AKT, IKK/NF-κB and ERK, resulting in the inducible transcription of genes that regulate the functions of activated B cells^1^. In addition, BCR crosslinking initiates an apoptosis programme for the elimination of auto-reactive B cells^2^. Thus, the coordinated regulation between apoptotic and BCR signalling controls both immune responses and homeostasis. However, the molecular switch that integrates the apoptotic pathway with BCR activation signalling remains unclear. In this regard, we speculated whether the onset of apoptosis following BCR activation could be modulated via crosstalk between post-translational modifications (PTMs) of critical apoptosis regulators.

*O*-GlcNAcylation is the addition of *O*-linked β-N-acetylglucosamine (*O*-GlcNAc) to serine/threonine residues on cytosolic and nuclear proteins. Two enzymes, *O*-GlcNAc transferase (OGT) and *O*-GlcNAcase (OGA), are responsible for the addition and removal of *O*-GlcNAc, respectively^3^. Altered regulation of *O*-GlcNAc modification has been found in diseases such as diabetes^4^ and Alzheimer's disease^5^. Because *O*-GlcNAcylation modifies proteins at serine/threonine residues that may overlap with the substrate sites of serine/threonine kinases, *O*-GlcNAcylation has been shown to play a role in modulating protein function by affecting protein phosphorylation. For example, competitive *O*-GlcNAcylation and phosphorylation at the S16 site of murine oestrogen receptor b mediates the degradation or stabilization of oestrogen receptor b^6^. In addition, *O*-GlcNAcylation of p53 at the S149 site, which abolishes phosphorylation at the adjacent T155 site, is critical for the ubiquitination and degradation of p53 and thereby results in p53 stabilization^7^. However, among the *O*-GlcNAcylated proteins identified thus far, only a few have been revealed to possess key regulatory and/or biological roles^8^.

In the immune system, it has been shown that the *O*-GlcNAcylation levels of nuclear proteins promptly increase during the early phase of T-lymphocyte activation, whereas *O*-GlcNAcylation on a portion of cytosolic proteins rapidly decreases^9^. Furthermore, nuclear factor of activated T cells (NFAT) and nuclear factor kappa-B (NF-κB) are modified by *O*-GlcNAcylation following the activation of either the Jurkat T or BJAB B-cell lines^10^, suggesting that, in addition to phosphorylation, protein *O*-GlcNAcylation may participate in the regulation of lymphocyte activation. However, the key regulatory proteins, their functional *O*-GlcNAcylation sites and the underlying significance of their crosstalk with other types of PTMs in B-cell activation remain unknown. To identify the molecular switch integrating BCR activation and the apoptosis pathway, we report the proteome-scale dissection of the functional interplay between protein *O*-GlcNAcylation and phosphorylation dynamics in activated primary B cells. We show that the inhibition of OGA promotes apoptosis in activated B cells, which is crucially dependent on the *O*-GlcNAcylation of lymphocyte-specific protein-1 (Lsp1), the recruitment of its kinase, PKC-β1, and the phosphorylation-mediated transduction of apoptotic signals.

## Results

### OGA inhibition promotes B-cell activation and apoptosis

**Figure 1:**
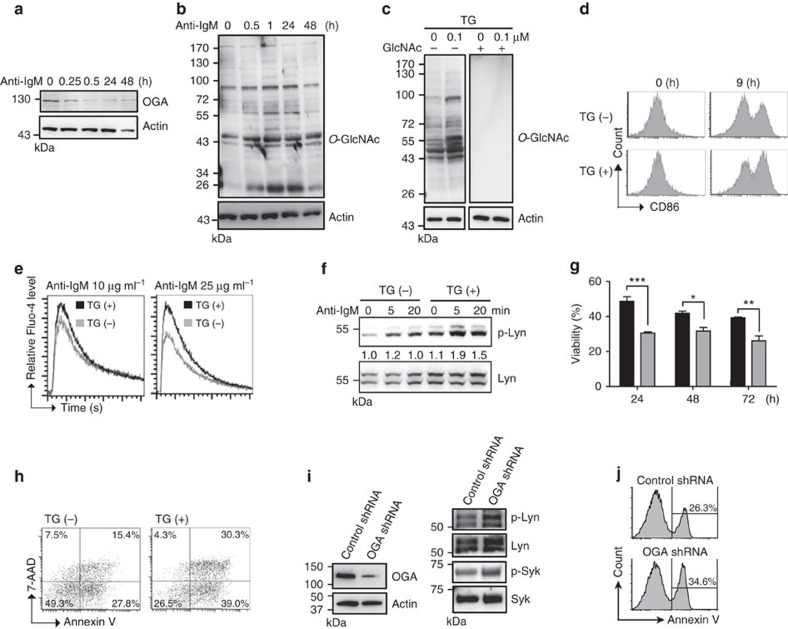
***O*****-GlcNAcylation accumulation as a result of TG treatment promotes B-cell activation and apoptosis.** (**a**) Immunoblotting (IB) showing the expression of OGA in mouse primary splenic B cells after anti-IgM (10 μg ml^−1^) stimulation at various time points. (**b**) IB showing the levels of *O*-GlcNAcylation after anti-IgM (10 μg ml^−1^) stimulation. (**c**) IB showing elevated levels of *O*-GlcNAcylated proteins after TG (1.0 μM) treatment of mouse splenic B cells. The specificity of anti-*O*-GlcNAc is validated in the presence of a 0.5 M GlcNAc competitor. (**d**) Flow cytometric analysis of surface CD86 expression showing the effect of pretreatment with TG (1.0 μM) for 8 h on the activation of mouse splenic B cells by anti-IgM (0.5 μg ml^−1^) treatment. (**e**) Levels of Ca^2+^ influx measured by Fluo-4 labelling after pretreatment with TG (1.0 μM) for 8 h and stimulation with indicated doses of anti-IgM. (**f**) IB showing increased levels of Lyn phosphorylation on tyrosine 397 in mouse splenic B cells that were pretreated with TG and stimulated with anti-IgM (10 μg ml^−1^) at the indicated time points. (**g**) Cell viability determined by trypan blue staining of splenic B cells that were pretreated with TG (shown as grey bar) and stimulated with anti-IgM (10 μg ml^−1^) for 24, 48 and 72 h. Results represent the mean±s.e.m. (*n*=3). *P<0.05, **P<0.01, ***P<0.001. (**h**) Flow cytometric analysis of Annexin-V and 7-AAD staining of splenic B cells pretreated with or without TG for 8 h and stimulated with anti-IgM (10 μg ml^−1^) for 48 h. (**i**) IB showing increased phosphorylation of Lyn (tyrosine 396) and Syk (tyrosine 525/526) in anti-IgM (10 μg ml^−1^) stimulated EGFP^+^ human Ramos B cells that were transfected with indicated shRNA for 24 h. (**j**) Flow cytometric analysis of the frequency of Annexin V^+^ Ramos B cells that were treated as described in **i** for 48 h.

Although it has been demonstrated that protein *O*-GlcNAcylation may play an important role in T- and B-cell activation^11^, the underlying mechanisms remain poorly understood. To further illuminate the mode of action of *O*-GlcNAc modification in B-cell activation, we purified mouse splenic B cells (Supplementary Fig. 1a) and demonstrated that the level of OGA was decreased in primary mouse splenic B cells beginning at 0.25 h after treatment with anti-IgM F(ab′)2 (Fig. 1a), which is consistent with the elevated levels of *O*-GlcNAcylation in splenic B cells after this treatment (Fig. 1b). There is also a dose-dependent effect of anti-IgM on the accumulation of *O*-GlcNAcylated proteins in splenic B cells (Supplementary Fig. 1b). However, the level of OGT was not changed after anti-IgM treatment (Supplementary Fig. 1c). We next treated mouse B cells with thiamet-G (TG), a specific inhibitor of OGA that causes increased *O*-GlcNAc modification^12^. As expected, immunoblotting showed that TG treatment increased *O*-GlcNAc levels and that *O*-GlcNAc immunoreactive signals were blocked after co-incubating anti-*O*-GlcNAc antibody with GlcNAc (Fig. 1c). By monitoring the expression of the activation surface marker CD86, we next examined the effects of TG treatment on B-cell activation caused by BCR crosslinking and found that treatment with TG caused modest increases in CD86 expression (Fig. 1d); this result was consistent with a previous work showing that pretreatment with PUGNAc, a less specific OGA inhibitor^13^, sensitizes B-cell activation^11^. Similarly, two other parameters that are used to indicate B-cell activation, the influx of Ca^2+^ and the phosphorylation of mouse Lyn at tyrosine 397 that represents the active form^14^, were also increased in the presence of TG (Fig. 1e,f). Notably, TG treatment of anti-IgM-stimulated B cells enhanced activation-induced cell death, as determined by the number of viable cells and an analysis of 7-AAD/Annexin V staining by flow cytometric analysis (Fig. 1g,h, respectively). Consistently, knockdown of OGA by shRNA potentiates anti-IgM-induced B-cell activation and apoptosis, as demonstrated by enhanced phosphorylation of human Lyn at tyrosine 396 and Syk at tyrosine 525/526 (Fig. 1i), and Annexin V^+^ frequency in stimulated Ramos B cells (Fig. 1j). Taken together, we found that the TG-induced accumulation of *O*-GlcNAc modifications enhanced B-cell activation and apoptosis after BCR crosslinking.

### *O*-GlcNAc-dependent phosphoproteome in activated B cells

We next analysed how *O*-GlcNAcylation perturbs phosphorylation-mediated B-cell activation/apoptosis and the potential interplay between *O*-GlcNAcylation and phosphorylation. The immunoblotting of mouse splenic B cells treated with TG was used to evaluate whether increased *O*-GlcNAcylation globally affects protein phosphorylation during B-cell activation. However, global changes in phosphorylation, as determined by immunoblotting with antibodies against pan-phosphoserine, phosphothreonine and phosphotyrosine, were not evident (Supplementary Fig. 2), suggesting that the accumulation of *O*-GlcNAcylation because of TG treatment may target specific phosphorylation events in stimulated B cells. Thus, a quantitative phosphoproteomic analysis was performed to globally identify site-specific phosphorylation changes in response to TG treatment. Mouse splenic B cells were treated with either anti-IgM (condition B) or TG before the anti-IgM treatment (condition C); untreated B cells were used as the control (condition A) (Supplementary Fig. 3a). The quantitative phosphoproteomic approach used in this study combined our previously developed automatic pH/acid-controlled IMAC procedure for phosphopeptide enrichment^15^ and the IDEAL-Q algorithm for label-free quantification^16^ (see workflow in Supplementary Fig. 3a).

A total of 3,752 non-redundant phosphopeptides that mapped to 3,079 unique phosphorylation sites originating from 1,266 proteins were identified (Supplementary Data 1). On the basis of the s.d. of our label-free quantitation strategy^17^, phosphopeptides showing more than twofold changes in abundance were considered to be altered phosphorylation sites with either upregulation or downregulation based on changes in either protein expression or phosphorylation stoichiometry^18^. Compared with the control (condition A), B-cell activation on anti-IgM stimulation (condition B) induced differential levels of 1,225 (32.6%) phosphopeptides from 632 phosphoproteins (B/A ratio). Furthermore, increased *O*-GlcNAc modification because of TG treatment altered 326 (8.7%) phosphopeptides, corresponding to 313 *O*-GlcNAcylation-dependent phosphosites from 224 phosphoproteins, in activated B cells (condition C/B), presumably representing subsets of the identified phosphoproteome sensitized to the effects of increased *O*-GlcNAcylation in activated B cells (Table 1).

**Figure 2:**
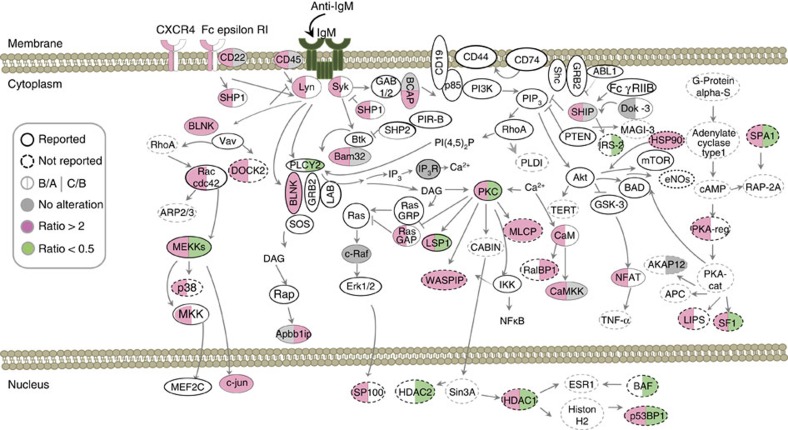
Perturbed protein phosphorylation in the BCR signalosome in response to TG treatment. The quantitative change in the levels of phosphopeptides in the BCR signalosome after anti-IgM treatment is indicated on the left half of the circle for each protein; the change from pretreatment with TG is shown on the right half. The colours red, green and grey represent overexpression (ratio >2), downregulation (ratio <0.5) or no change in the phosphorylation site of each protein, respectively. Sample A represents B cells without treatment. Sample B represents activated B cells on treatment with anti-IgM (10 μg ml^−1^) for 10 min; sample C was also pretreated with TG (0.1 μM) for 8 h. Circles with solid lines represent known molecules in BCR signalling, while those depicted in dashed lines have not been reported in this pathway but are added through using Ingenuity Pathways Analysis and literature mining (CD45 is PTPRC, BCAP is Pik3ap1, SHIP is Inpp5D, PKC is PRKC and Bam32 is DAPP1).

Next, we focus on targets associated with BCR signalling. In Fig. 2, we illustrate the differential phosphorylation of 22 previously characterized proteins involved in the BCR signalling pathway (depicted in circles with a solid line), including Lyn, Syk, SHP1 and BLNK, as well as proteins in the PLC-γ2/calcium/NFAT, PI3K, IKK/NF-κB and ERK pathways. Through using a web-based bioinformatics software, Ingenuity Pathways Analysis, and literature mining, we further identified 17 phosphoproteins from our proteomics results that are not reported to be involved in the BCR signalling pathway but are closely related to the downstream molecules of BCR signalling, including Dock-2, insulin receptor substrate (IRS-2) and HDAC-1 (circles with a dashed line). The absence of a few known molecules in BCR signalling, including Bruton's tyrosine kinase and PI3K, may be a result of the prompt transfer of phosphate groups or the limited detection sensitivity of current proteomics technology.

On the basis of the quantitation results, we filtered potential targets of *O*-GlcNAcylation–phosphorylation interplay in BCR signalling. Ideally, such molecules exhibit elevated phosphorylation in response to anti-IgM stimulation but display altered phosphorylation levels in response to TG pretreatment. Using Nop56 as an example, TG pretreatment reduced Nop56 phosphorylation at S513 and S543 (Supplementary Fig. 3b). Site-specific alteration was observed for Map4k1: enhanced *O*-GlcNAcylation suppressed two phosphorylation sites, S452 and S455, whereas phosphorylation at S373/S375 was not affected, and S419 showed slightly increased phosphorylation levels compared with that under basal-level *O*-GlcNAcylation (Supplementary Fig. 3c). In summary, among the 44 identified phosphoproteins in BCR signalling, as many as 15 (34.1%) phosphoproteins showed altered phosphorylation levels, suggesting that *O*-GlcNAcylation exerts global effects on the dynamic phosphorylation-mediated BCR signalosome. Site-specific alterations also indicated that the crosstalk between *O*-GlcNAcylation and phosphorylation may alternatively occur at the same sites of a given protein but may not be a general event.

### Temporal *O*-GlcNAcylation and phosphorylation on Lsp1

**Figure 3:**
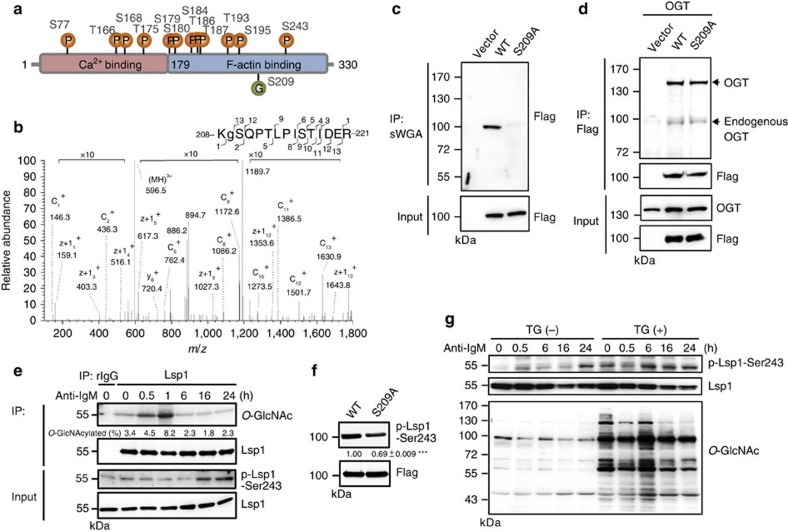
**Dynamic interplay between** ***O*****-GlcNAcylation and phosphorylation on Lsp1 in B cells after anti-IgM stimulation**. (**a**) Mass spectrometric results revealing 12 mapped phosphorylation sites and 1 *O*-GlcNAcylation site on Lsp1. (**b**) Mapping of the *O*-GlcNAcylation site 208-KSQPTLPISTIDER-221 of Lsp1 using ETD fragmentation during MS/MS analysis. The ions, c_1_^+^ (146.3 Da), c_2_^+^ (436.3 Da), z+1_12_^+^ (1353.5 Da), z+1_13_^+^ (1643.8 Da) suggest that S209 is *O*-GlcNAcylated. (**c**) Lysates prepared from Ramos B cells overexpressing the vector control, Flag-EGFP-tagged WT or S209A Lsp1 were subjected to a pull-down assay using sWGA agarose beads, followed by immunoblotting (IB) with an anti-Flag antibody. (**d**) Lysates from 293T cells ectopically expressing OGT and either vector, Flag-EGFP-tagged WT or S209A Lsp1, were used for immunoprecipitation (IP) with anti-Flag, followed by IB with the indicated antibodies. (**e**) IB showing the levels of Lsp1, S243 phosphorylated Lsp1 and *O*-GlcNAcylated Lsp1 in anti-IgM (10 μg ml^−1^) stimulated mouse splenic B cells at indicated time points in an IP assay with control rabbit IgG (rIgG) or anti-Lsp1-specific antibody crosslinked to protein A agarose. The percentage of *O*-GlcNAcylated Lsp1 is indicated. (**f**) Sorted EGFP^+^ Ramos B cells overexpressing Flag-EGFP-tagged WT or S209A Lsp1 were stimulated with anti-human IgM (25 μg ml^−1^) for 30 min, followed by IB analysis of Lsp1 S243 phosphorylation. One representative experiment out of three is shown. The relative levels of Lsp1 with phosphorylation at S243 were indicated. Results represent the mean±s.e.m. (*n*=3). ***P<0.001. (**g**) IB showing the levels of Lsp1 S243 phosphorylation in mouse splenic B cells after pretreatment with TG for 8 h and anti-IgM (10 μg ml^−1^) stimulation at the indicated time points.

Among the 15 proteins in the BCR signalling pathway that showed potential interplay between *O*-GlcNAcylation and phosphorylation, Lsp1 drew our attention because this protein is known as a pro-apoptotic regulator in anti-IgM-induced apoptosis^19^ even though the underlying mechanisms are unclear. Thus, we further investigated whether the site-specific *O*-GlcNAcylation of Lsp1 interferes with its phosphorylation and plays a role in anti-IgM-induced apoptosis. Here we have identified 17 unique phosphopeptides of Lsp1, including 12 phosphorylation sites (S77, T166, S168, T175, T179, S180, S184, T186, T187, T193, S195 and S243; Fig. 3a). All of them were known phosphorylation sites except for T193. Sites that are sensitive to TG treatment were illustrated (Supplementary Fig. 4a). We then validated that Lsp1 was present in anti-*O*-GlcNAc immunoprecipitates from the lysates of BCL-1 cells, a murine B-cell lymphoma line (Supplementary Fig. 4b). Reciprocally, *O*-GlcNAc-modified proteins were detected in anti-Lsp1 immunoprecipitates (Supplementary Fig. 4c). To definitively map *O*-GlcNAc sites on Lsp1, vectors expressing Flag-EGFP-tagged Lsp1 and OGT were co-transfected into human embryonic kidney 293T cells. Lsp1 that was purified by anti-Flag was subjected to both collision-induced dissociation (CID) and electron-transfer dissociation (ETD) tandem mass spectrometry (MS/MS) to identify *O*-GlcNAcylation sites. *O*-GlcNAcylation-modified peptides of Lsp1 (^208^KSQPTLPISTIDER^221^) were observed at [M+3H]^3+^ *m*/*z* 596.66, and its *O*-GlcNAcylation at S209 was confirmed by both ETD- and CID-fragmentation mass spectra (Fig. 3b and Supplementary Fig. 4d).

Site-directed mutagenesis was performed to generate a mutant Lsp1 in which amino acid 209 is replaced by Ala (S209A). Lysates from Ramos B cells transfected with vectors expressing either Flag-EGFP-tagged wild-type (WT) or S209A Lsp1 were subjected to a pull-down assay using succinylated wheat germ agglutinin (sWGA), which is a lectin that has high affinity for *O*-GlcNAc (ref. 20). The results shown in Fig. 3c indicate that compared with WT Lsp1, S209A Lsp1 can only be slightly pulled down by sWGA, suggesting that S209 is the major *O*-GlcNAc site on Lsp1. We further confirmed that the lower *O*-GlcNAcylation level found on S209A Lsp1 was not a result of an impaired interaction with OGT because nearly equal amounts of OGT were immunoprecipitated by WT and S209A Lsp1 (Fig. 3d). During anti-IgM stimulation in splenic B cells, kinetic measurements indicated that Lsp1 *O*-GlcNAcylation, as detected by immunoprecipitation with anti-Lsp1 antibody crosslinked to protein A agarose, occurred rapidly and peaked at 1 h after stimulation (Fig. 3e, upper panel). Likewise, sWGA pull-down assay also showed similar kinetics of Lsp1 *O*-GlcNAcylation in stimulated B cells (Supplementary Fig. 4e). Among the 12 identified phosphorylation sites of Lsp1, site S243 is upregulated most markedly on TG treatment (Supplementary Fig. 4a). Therefore, Lsp1 phosphorylation was examined using a commercially available antibody against Lsp1 phosphorylated at S243. Following anti-IgM stimulation, S243 phosphorylation was continuously augmented at 16–24 h (Fig. 3e, lower panel); this occurred later than the rapid rise in *O*-GlcNAcylation. More importantly, the Lsp1 mutation S209A significantly reduced S243 phosphorylation in anti-IgM-stimulated Ramos B cells (Fig. 3f), showing that Lsp1 *O*-GlcNAcylation at the S209 site is required for the phosphorylation of S243. Consistently, after BCR crosslinking, the phosphorylation of Lsp1 at S243 was gradually enhanced in TG-treated splenic B cells at various time points after anti-IgM stimulation (Fig. 3g). Furthermore, TG treatment in stimulated B cells resulted in the *O*-GlcNAcylation of Lsp1 at earlier time points, which is correlated with the earlier induction of phosphorylation of Lsp1 at S243 (Supplementary Fig. 4f).

### Apoptosis requires Lsp1 *O*-GlcNAcylation and phosphorylation

**Figure 4:**
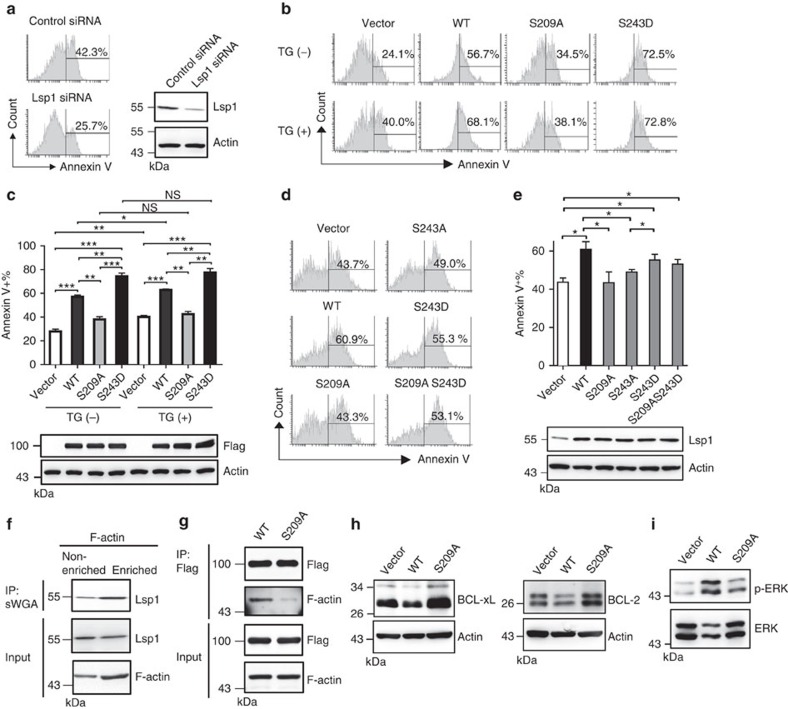
***O*****-GlcNAcylation of Lsp1 controls B-cell apoptosis by enhancing ERK phosphorylation and reducing BCL-2 and BCL-xL expression.** (**a**) Flow cytometric analysis of the frequency of Annexin V^+^ Ramos B cells transfected with control siRNA or Lsp1-specific siRNA, and stimulated with anti-IgM (25 μg ml^−1^) for 48 h. Immunoblotting (IB) showing the knockdown of endogenous Lsp1 by Lsp1 siRNA. (**b**) Flow cytometric analysis of the frequency of Annexin V^+^ cells among EGFP^+^ Ramos B cells lentivirally transduced with the indicated vectors and stimulated with anti-IgM (25 μg ml^−1^) with or without the treatment with TG for 48 h. (**c**) Statistical results of the Annexin V^+^ cells described in **b** are shown. IB showing the equal expression of various variants of Flag-EGFP-tagged Lsp1. (**d**) The frequency of Annexin V^+^ cells in the YFP^+^ gate of mouse splenic B cells transduced with the indicated retroviral vectors and stimulated with anti-IgM (10 μg ml^−1^) for 48 h. (**e**) Statistical results of the Annexin V^+^ cells described in **d** are shown. IB showing the equal expression of various variants of Flag-tagged Lsp1. The results in **c**,**e** represent the mean±s.e.m. (*n*=3). *P<0.05, **P<0.01, ***P<0.001. (**f**) F-actin enrichment was performed by biotin-phalloidin pull-down with streptavidin-agarose beads. IB showing the levels of endogenous *O*-GlcNAcylated Lsp1, as pulled down by sWGA, in the F-actin-enriched and non-enriched compartments from lysates of Ramos B cells stimulated with anti-IgM (25 μg ml^−1^) for 30 min. (**g**) Lysates from sorted EGFP^+^ Ramos B cells ectopically expressing Flag-EGFP-tagged WT or S209A Lsp1 and stimulated with anti-IgM (25 μg ml^−1^) for 30 min were used for IP with anti-Flag, followed by IB with the indicated antibodies. (**h**) IB showing the expression of BCL-2 and BCL-xL in sorted EGFP^+^ Ramos B cells transduced with the indicated vectors and stimulated with anti-IgM (25 μg ml^−1^) for 48 h. (**i**) IB showing the phosphorylation of ERK in the cells described in **h**.

Lsp1 appears to be crucial for the induction of apoptosis in B cells after IgM stimulation because knockdown of Lsp1 reduced the frequency of anti-IgM-induced apoptosis in Ramos B cells (Fig. 4a). To further examine the biological outcome of the interplay between the *O*-GlcNAcylation and phosphorylation of Lsp1 in B-cell activation, we ectopically expressed Flag-EGFP-tagged WT or various mutated Lsp1 variants using a lentiviral vector in Ramos B cells and Flag-tagged WT or various variants of Flag-tagged Lsp1 via a retroviral vector in splenic B cells (Fig. 4c,e). As reported previously^19^, ectopic expression of WT Lsp1 significantly enhanced anti-IgM-induced B-cell apoptosis in Ramos B cells and mouse primary splenic B cells, as determined by Annexin V staining (Fig. 4b–e). However, Lsp1 did not affect B-cell activation (Supplementary Fig. 5a). Remarkably, ectopic expression of S209A Lsp1 induced much less apoptosis in Ramos B cells compared with WT Lsp1 (Fig. 4b,c). Mutation with an aspartic acid at the 243 site of Lsp1 (S243D), which acts as a phospho-mimicry mutation, resulted in enhanced anti-IgM-induced apoptosis compared with WT Lsp1 (Fig. 4b,c). TG treatment caused increased levels of apoptosis after anti-IgM stimulation in Ramos B cells carrying vector alone or exogenous WT Lsp1 (Fig. 4b,c). In contrast, TG did not enhance apoptosis in S209A Lsp1-expressing Ramos B cells (Fig. 4b,c). This result is consistent with another line of finding that TG did not increase the phosphorylation at S243 on S209A Lsp1 (Supplementary Fig. 5b). Likewise, S209A Lsp1-expressing mouse splenic B cells showed reduced anti-IgM-induced apoptosis (Fig. 4d,e). Mutating serine to alanine at the 243 site of Lsp1 (S243A) reduced anti-IgM-induced apoptosis, while ectopic expression of S243D resulted in enhanced apoptosis compared with S243A that is similar to the effect caused by the double mutant S209A and S243D (Fig. 4d,e). These combined results suggest that *O*-GlcNAcylation at the Lsp1 S209 site and *O*-GlcNAcylation-dependent phosphorylation at the S243 site contribute to anti-IgM-induced apoptosis.

To further understand why the *O*-GlcNAcylation of Lsp1 at S209 potentiates anti-IgM-induced apoptosis, we monitored the expression of CD95, which controls extrinsic apoptotic B-cell signalling^21^, in S209A and WT Lsp1-expressing anti-IgM-stimulated splenic B cells. However, CD95 levels were not affected when the *O*-GlcNAc site was mutated (Supplementary Fig. 5c). Given that Lsp1 was shown to translocate to the cytoplasmic side of the B-cell plasma membrane after anti-IgM treatment^22^ and that Lsp1 binds with F-actin^23^, the polymerization of which contributes to BCR crosslinking-induced apoptosis^24^, we suspected that the F-actin-mediated translocation of Lsp1 and apoptosis may be coordinately controlled by Lsp1 *O*-GlcNAcylation. Indeed, higher level of *O*-GlcNAcylated Lsp1 was detected in the F-actin-enriched fraction that was pulled down by phalloidin^25^ (Fig. 4f). Moreover, much less F-actin was co-immunoprecipitated with Flag-EGFP-tagged S209A Lsp1 in lysates from Ramos transfectants as compared with Flag-EGFP-tagged WT Lsp1 (Fig. 4g). We next examined the expression of BCL-2 and BCL-xL, two important anti-apoptotic molecules that protect against anti-IgM-induced apoptosis^26^,^27^, and found that their levels were reduced in cells expressing WT Lsp1, but not in S209A, compared with control vector-transduced cells (Fig. 4h). Lsp1-mediated apoptosis is associated with the activation of ERK kinase^28^, and we found that the overexpression of WT Lsp1 caused significant increases in ERK phosphorylation in anti-IgM-stimulated B cells; in contrast, S209A-expressing cells only exhibited modest increases in ERK phosphorylation (Fig. 4i). Together, these data suggest that following anti-IgM stimulation, *O*-GlcNAcylation at S209 of Lsp1 causes increased ERK phosphorylation and the reduced expression of BCL-2 and BCL-xL, leading to the apoptosis of activated B cells.

### Lsp1 *O*-GlcNAcylation recruits PKC-β1 to induce apoptosis

**Figure 5:**
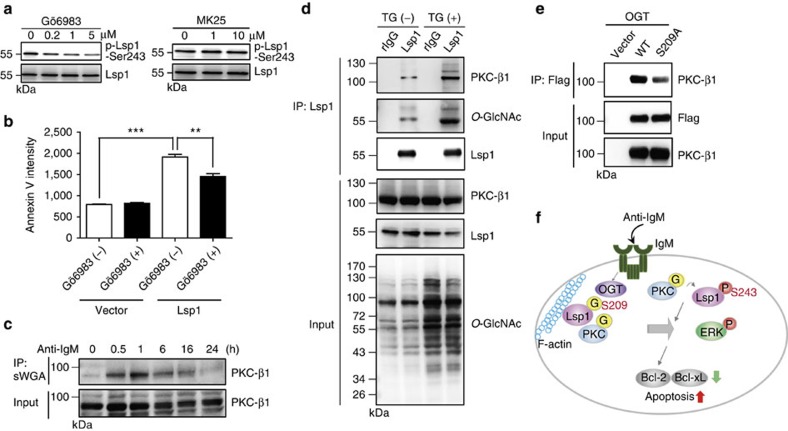
***O*****-GlcNAcylation of Lsp1 affects the recruitment of PKC-β1.** (**a**) Immunoblotting (IB) showing the effects of various doses of a PKC inhibitor, Gö6983 (left panel), or an MK2 inhibitor, MK25 (right panel), on the levels of Lsp1 S243 phosphorylation in anti-IgM (10 μg ml^−1^)-stimulated mouse splenic B cells. Inhibitors were added for 1 h, and the cells were collected 24 h after anti-IgM stimulation. (**b**) Statistical results (mean±s.e.m., *n*=3) showing the mean fluorescent intensity of Annexin V staining from the flow cytometric analysis of splenic B cells expressing the indicated vectors and stimulated with anti-IgM (10 μg ml^−1^) in the presence or absence of Gö6983 at 24 h. **P<0.01, ***P<0.001. (**c**) IB showing the kinetics of PKC-β1 *O*-GlcNAcylation in anti-IgM (10 μg ml^−1^)-stimulated mouse splenic B cells by sWGA pull-down. (**d**) Lysates from Ramos B cells pretreated with or without TG (1.0 μM) for 8 h were subjected to IP with either control rabbit Ig (rIg) or anti-Lsp1, followed by IB with the indicated antibodies. (**e**) Lysates prepared from 293T cells expressing Flag-EGFP-tagged WT or S209A Lsp1 were used for IP with anti-Flag, followed by IB with the indicated antibody. (**f**) Model of the role of Lsp1 in anti-IgM-induced apoptosis. Following antigen stimulation, OGT interacts with Lsp1 and catalyses the *O*-GlcNAcylation of Lsp1 at S209. PKC-β1 is also *O*-GlcNAcylated. *O*-GlcNAcylation of Lsp1 enhances the recruitment to F-actin compartment and the interaction with PKC-β1, causing its subsequent phosphorylation at S243, which in turn activates ERK and downregulates the anti-apoptotic proteins BCL-2 and BCL-xL.

Given that S209 *O*-GlcNAcylation mediates S243 phosphorylation and promotes anti-IgM-induced apoptosis, we sought to further delineate the underlying molecular mechanisms that link the effects of S209 *O*-GlcNAcylation and S243 phosphorylation. In B cells, Lsp1 is a substrate of PKC^29^ and recruits PKC-β1 to enforce apoptosis^28^; Lsp1 is also phosphorylated by MAPKPKA kinase (MK2) at the S243 site in neutrophils^30^. To identify which kinase contributes to the phosphorylation of Lsp1 at S243, primary mouse splenic B cells were pretreated with MK25, an MK2 kinase inhibitor, or Gö6983, a PKC inhibitor. We found that treatment with Gö6983 dose-dependently reduced Lsp1 phosphorylation at S243 (Fig. 5a, left panel), but treatment with MK25 did not reduce phosphorylation (Fig. 5a, right panel). Treatment with Gö6983 also attenuated the pro-apoptotic effects of WT Lsp1 on anti-IgM-induced apoptosis (Fig. 5b). These data suggest that PKC-β1 mediates the phosphorylation of Lsp1 at S243, which may result in enhanced anti-IgM-induced apoptosis. In agreement with the observation that PKC-β1 is *O*-GlcNAcylated in rat hepatocytes, PKC-β1 was also found to be rapidly *O*-GlcNAcylated, peaking at 1 h after BCR crosslinking in B cells, as detected by sWGA pull-down (Fig. 5c). Because PKC-β1 regulates the S243 phosphorylation of Lsp1 and *O*-GlcNAcylation at S209 affects S243 phosphorylation, we assessed whether *O*-GlcNAcylation at S209 modulates the recruitment of PKC-β1 to phosphorylate Lsp1 at S243. We examined the amounts of PKC-β1 binding to Lsp1 in the absence or presence of TG. Of note, the elevated *O*-GlcNAc modification of Lsp1 by TG treatment resulted in increased interactions with PKC-β1 because more PKC-β1 was detected in anti-Lsp1 immunoprecipitates when using the lysates of TG-treated cells (Fig. 5d). Accordingly, we found that less PKC-β1 interacts with S209A Lsp1 compared with WT Lsp1 (Fig. 5e). These data suggest that *O*-GlcNAc modification at S209 of Lsp1 promotes the recruitment of PKC-β1.

## Discussion

*O*-GlcNAcylation has been shown to be involved in several cellular events and to regulate many important cellular functions. However, the role of *O*-GlcNAcylation in immune cells at the molecular level has not been extensively studied to date. B cells rapidly increase glucose uptake and glycolysis during the early phase of BCR engagement^31^. We found that global *O*-GlcNAcylation is dynamically altered after BCR crosslinking, which may reflect the rapid uptake and utilization of glucose that can be used as the source for UDP-GlcNAc synthesis via the hexosamine biosynthetic pathway and for *O*-GlcNAc modification via OGT. Our results therefore imply that glucose acquisition and metabolism may contribute to the regulation of antigen-induced B-cell activation and apoptosis.

Treatment with PUGNAc has been shown to sensitize lymphocyte activation^11^. PUGNAc is an inhibitor of a variety of *N*-acetylhexosaminidases, and its specificity for OGA is 35,000-fold less than that of TG^32^. In our study, we found that *O*-GlcNAcylation accumulation resulting from TG treatment not only potentiates B-cell activation but also promotes anti-IgM-induced apoptosis. Although our mass spectrometry results did not reveal the potential *O*-GlcNAcylation and phosphorylation interplay of NF-kB and NFAT, the *O*-GlcNAcylation of which was previously reported to be important for controlling B-cell activation^10^, our data did suggest other TG-responsive molecules that are known to be critical to B-cell activation and apoptosis, such as PKC, PLC-γ2 and Lsp1. We also identified the mediator, PKC-β1, as the kinase of Lsp1 that links its site-specific *O*-GlcNAc and phosphorylation. The *O*-GlcNAcylation of Lsp1 at S209 is required to bind to PKC-β1, thereby inducing the phosphorylation of Lsp1 at S243. It is still obscure why there is a time lag between the peaks of *O*-GlcNAcylation and S243 phosphorylation of Lsp1 as our data showed that *O*-GlcNAcylation of Lsp1 peaks at 1 h and the phosphorylation of Lsp1 at S243 occurs at later time points. We suspect this could be due to changes in the cellular localization of Lsp1. Supporting this notion, we found that *O*-GlcNAcylation of Lsp1 promotes the interaction with F-actin and its recruitment to the F-actin compartment. A previous study reported that Lsp1 S243 is involved in neutrophil polarization^30^; however, the role of Lsp1 phosphorylation at the S243 site in cell survival has not yet been reported. We here show that phosphorylation of Lsp1 at S243 promotes anti-IgM-induced apoptosis in B cells. Taken together, it is conceivable that, on antigen ligation of BCRs, *O*-GlcNAcylated Lsp1 and PKC-β1 translocate to the plasma membrane of B cells, resulting in PKC-β1-mediated increased levels of Lsp1 phosphorylation at S243, which causes the activation of a signalling complex containing ERK and apoptosis (Fig. 5f).

In previous studies, protein *O*-GlcNAcylation was proposed to antagonize the effects of protein phosphorylation because both modifications occur at Ser/Thr sites. Accumulating evidence also suggests other types of interplay between *O*-GlcNAcylation and phosphorylation, such as reciprocal modification^33^. In the present study, quantitative phosphoproteomic results revealed 8.7% alteration in the levels of total phosphorylation of identified phosphopeptides on accumulated *O*-GlcNAcylation. We also found that PKC is *O*-GlcNAcylated after BCR crosslinking. However, whether OGT, PKC-β1 and Lsp1 form a complex after B-cell activation remains unknown. It is notable that PKC-β family proteins are indispensable for BCR-induced NF-κB activation and survival signalling^34^. Therefore, it is plausible that PKC-β1 may be a double-edged sword in promoting or preventing apoptosis during B-cell activation, depending on the status of its interactions with Lsp1.

The expression of Lsp1 has been shown to be of diagnostic value for some lymphoid tumours, such as Hodgkin's disease^35^. Moreover, Lsp1 is aberrantly expressed in hairy cell leukaemia^36^. Our results revealed that PTMs of Lsp1 by *O*-GlcNAcylation and phosphorylation may affect its function and the survival of activated primary B cells. Whether such PTM interplay exists in B-cell malignancies and can be applied to control treatment outcomes warrants further study. In summary, we demonstrate the temporal regulation among Lsp1 *O*-GlcNAcylation, its kinase recruitment and *O*-GlcNAcylation-dependent phosphorylation, which coordinately transduces apoptotic signals in activated B cells.

## Methods

### Animals and cell culture

Six- to eight-week-old C57BL/6 mice were purchased from the National Laboratory Animal Center in Taiwan. The animal experimental protocol was approved by the Institutional Animal Care and Utilization Committee at Academia Sinica. Ramos B cells from Bioresource Collection and Research Center, Taiwan (BCRC) (BC-60252) and BCL-1 cells (ATCC, CRL-1669) were cultured in RPMI 1640 medium (Life Technologies) containing 10% fetal bovine serum (FBS), 100 U ml^−1^ penicillin and 100 μg ml^−1^ streptomycin (Life Technologies) at 37 °C with 5% CO_2_. 293T (American Type Culture Collection, CRL-3216) and 3T3 cells (BCRC, BC-60071) were maintained in DMEM (Dulbecco's modified Eagle's medium, Life Technologies) containing 10% FBS, 100 U ml^−1^ penicillin and 100 μg ml^−1^ streptomycin at 37 °C with 5% CO_2_. Cell lines used in this study are free from mycoplasma contamination as examined by using EZ-PCR mycoplasma test kit (Biological Industries, 20-700-20). Cell viability was monitored by trypan blue staining. Mouse splenic B cells were purified using anti-B220 microbeads (Miltenyi Biotec) from 6- to 8-week-old C57BL/6 mice and cultured in RPMI 1640 medium supplemented with 10% charcoal/dextran-treated FBS, 100 U ml^−1^ penicillin, 100 μg ml^−1^ streptomycin and 50 μM 2-ME at 37 °C with 5% CO_2_. For mouse splenic B-cell stimulation, purified B cells at 2 × 10^6^ cells per ml were treated with goat-anti-mouse IgM F(ab′)2 (Jackson ImmunoResearch Laboratories). For blocking OGA activity, TG (0.1 μM, Cayman) was used to pretreat mouse splenic B cells or Ramos B cells 8 h before anti-IgM stimulation and was re-added into cell culture 1 day later. In some experiments, an MK2 inhibitor, MK25, or a PKC inhibitor, Gö6983, both purchased from Cayman, were added 1 h before anti-IgM stimulation. In some experiments, Ramos B cells were stimulated with a goat anti-human IgM F(ab′)2 fragment (Jackson ImmunoResearch Laboratories).

### Retroviral and lentiviral transduction

To ectopically express mouse Lsp1 (mLsp1) or mouse OGT (OGT) in mammalian cells, a cDNA encoding full-length mLsp1 or OGT was cloned by RT–PCR from mouse splenic B cells. mLsp1 was then cloned into the pEGFP-N1 expression vector (BD Biosciences Clonetech), and OGT was cloned into the pFlag-CMV2 expression vector (Sigma). The detailed cloning procedures for the site-directed mutagenesis of various mLsp1 mutants, including S209A, S243A, S243D and S209AS243D, are available on request. For retroviral or lentiviral transduction, various forms of mLsp1, including WT and mutants, were cloned into the pGC-YFP retroviral vector^37^ or pFUGW lentiviral vector^38^. The detailed cloning procedures are available on request. The procedures for the production of retroviral or lentiviral vector were as described^38^. Mouse splenic B cells or B-cell lines were transduced with viral vectors at a multiplicity of infection of 1–5 in the presence of 5 μg ml^−1^ polybrene (Sigma). YFP^+^ or GFP^+^ cells were examined or sorted using a FACSCanto or FACSAria (Becton Dickinson) at 48 or 72 h after transduction.

### Flow cytometry and calcium flux

The procedure for the flow cytometric analysis was performed as described^37^. The antibodies used in this study were as follows: anti-phycoerythrin-conjugated anti-mouse CD86 (clone GL1; BD PharMingen) and anti-phycoerythrin-Cy7 (PE-Cy7)-conjugated CD95 (clone Jo2, BD PharMingen). The cells were analysed using a FACSCanto (Becton Dickinson) with FACS Express 3.0 software. For detecting apoptotic cells, Annexin V and 7-AAD staining (BD PharMingen) were performed according to the manufacturer's protocols. The levels of calcium flux after anti-IgM stimulation were examined by labelling primary splenic B cells with Fluo-4AM dye (Invitrogen, 1 μM) at a density of 2 × 10^6^ cells per ml in RPMI for 30 min at room temperature. The labelled cells were then washed and resuspended in Hank's balanced salt solution and stimulated by anti-IgM (25 or 10 μg ml^−1^) immediately before FACS analysis using a FACSCalibur (Becton Dickinson).

### Protein purification and analysis

Protein analysis, including immunoprecipitation and immunoblotting, was performed as described^39^. Briefly, mouse splenic B cells were lysed in IP lysis buffer containing 50 mM Tris-Cl (pH 8.0), 150 mM NaCl, 5 mM EDTA, 0.5% TritonX-100, 0.1% sodium deoxycholate, protease and phosphatase inhibitor cocktail (Roche) and 10 nM TG, followed by incubation on ice for 30 min. Samples were then centrifuged at 12,000 r.p.m. at 4 °C for 15 min to collect supernatant, which was then further pre-cleared by protein A beads (Santa CruZ, sc-2001). A unit of 5 μg of anti-Lsp1 antibody (Abcam, ab21637), anti-Flag antibody (Sigma, F3165), anti-*O*-GlcNAc antibody (clone: RL-2, Abcam, ab2739), rabbit IgG (Bethyl, p120-101) or mouse IgG (Santa-CruZ, sc-2025) were added to and incubated with the pre-cleared lysates at 4 °C for overnight, followed by incubation with protein A beads. Primary antibodies used in immunoblotting are anti-*O*-GlcNAc (clone: CTD110.6, Sigma, O77764, 1:1,000), Lsp1 (Abcam, ab21637, 1:200; Santa CruZ, sc23804, 1:200), phospho-Ser243 Lsp1 (Abcam, ab21636, 1:1,000), ERK (Cell Signaling, #4695, 1:1,000), phospho-Thr202/Tyr204 ERK (Cell Signaling, #4370,1:1,000), PKC-β1 (Avivasysbio, ARP56423, 1:1,000), OGT (GeneTex, GTX30868, 1:1,000), OGA (Santa CruZ, sc-66612, 1:1,000), BCL-2 (CALBIOCHEM, #OP6D, 1:250), BCL-xL (Santa CruZ, sc-23958, 1:250), pan-Serine phosphorylation (GeneTex, GTX26639, 1:1,000), pan-threonine phosphorylation (Cell Signaling, #9386, 1:1,000), pan-tyrosine phosphorylation (Millipore, 05-321, 1:1,000), phospho-Lyn (Tyrosine 396) (Abcam, ab40660, 1:1,000), Lyn (Biolegend, 628102, 1:1,000), phospho-Syk (Tyrosine 525/526) (Cell Signaling, 2710S, 1:500), Syk (Abcam, ab53777, 1:500), F-actin (Abcam, ab130935, 1:200) and Flag antibody (Sigma, F3165, 1:1,000). Secondary antibodies used in immunoblotting are goat anti-mouse IgG horseradish peroxidase (HRP)-conjugated antibody (Sigma, A2554, 1:5,000), goat anti-rabbit IgG HRP-conjugated antibody (Sigma, A0545, 1:5,000) and rabbit anti-goat IgG HRP-conjugated antibody (Sigma, A5420, 1:5,000). In some immunoblotting used after immunoprecipitation, goat anti-mouse IgG light chain-specific antibody conjugated with HRP (Millipore, AP200P, 1:5,000) and mouse anti-rabbit IgG light chain-specific antibody conjugated with HRP (Millipore, MAB201, 1:500) were used. The immunoreactive proteins are detected by Western Bright Sirius chemiluminescent detection reagent (Advansta, R-030027-C50) according to the manufacturer's instruction. Chemiluminescent signal images were captured using the LAS 3,000 system (Fujifilm). All immunoblotting experiments were performed at least twice and the representative images were cropped for presentation. Uncropped scanned blots are presented in Supplementary Figs 6−10.

To perform purification of full-length mLsp1 for mass spectrometric analysis, expression vector encoding Flag-EGFP-mLsp1 was transfected to 293T cells for 48 h, and the recombinant Flag-EGFP-Lsp1 proteins were then collected and purified by anti-Flag antibody using previously described protocols^40^. To pull down *O*-GlcNAcylated Lsp1 and PKC-β1, 50 μl sWGA agarose (Vector Laboratories, AL-1023S) were added to cell lysate and incubated at 4 °C for 12 h, and washed five times with IP lysis buffer. Finally, *O*-GlcNAcylated proteins were eluted by 5 × sample buffer containing 250 mM Tris-Cl (pH 6.8), 4% bromophenol blue, 2% SDS, 50% glycerol and 10% β-mercaptoethanol at 95 °C for 5 min for following immunoblotting analysis. For detection of *O*-GlcNAcylated levels of endogenous Lsp1 in primary mouse splenic B cells after anti-IgM stimulation, 15 μg anti-Lsp1 antibody (Santa CruZ, sc-23804) was crosslinked to 30 μl protein A agarose by disuccinimidyl suberate and immobilized to a spin column according to the manufacturer's instructions (Thermo Fisher Scientific, 26147). After crosslinking, 250 μg stimulated cell lysates were added to the antibody/agarose complex in a spin column, and incubated at 4 °C for 12 h. Purified Lsp1 protein was obtained by washing the column five times with IP lysis buffer and then eluted with 50 μl elution buffer (Thermo Fisher Scientific, 26147).

### F-actin enrichment

In all, 3 × 10^7^ Ramos B cells were lysed in 400 μl IP lysis buffer and subjected to immunoprecipitation with 50 μg biotin-phalloidin (Sigma, P8716) at room temperature for 12 h, followed by incubation with 50 μl streptavidin agarose resin (Thermo Fisher Scientific, 20347) at room temperature for 2 h and then spin-down at 1,000*g* at 4 °C for 1 min. The supernatant was collected and used as the F-actin non-enriched fraction. Biotin-phalloidin-/streptavidin agarose-bound proteins were resuspended in 400 μl IP lysis buffer and then used as the F-actin-enriched fraction. To detect the *O*-GlcNAcylation levels of Lsp1 within each fraction, 50 μl sWGA agarose (Vector Laboratories, AL-1023S) was added as described above.

### siRNA and shRNA transfection

Transfection of siRNAs (purchased from BIOTOOLS) and the plasmid expressing shRNA (from National RNAi core Facility, Academia Sinica, Taipei, Taiwan) was performed by using 3 × 10^6^ Ramos B cells and Lonza Amaxa Nucleofector V kit according to the manufacturer's suggestions. A unit of 300 nM siRNAs or 3 μg shRNA expression plasmids were mixed with cells/Nucleofector solution and the electroporation was carried out according to the protocols provided by Nucleofector instrument. The sequences of siRNA and shRNA are as follows: Lsp1 siRNAs: mixture of 5′-GGACAAAGAUAAAGAAGAUTT-3′, 5′-GUCCUCUGAACUGGAUGAATT-3′ and 5′-CUGGAGACAUGAGCAAGAATT-3′; control siRNA: 5′-UUCUCCGAACGUGUCAUGUTT-3′; OGA shRNA: 5′-GAGCTCATCCCACGGTTAAAG-3′; and control shRNA targeting to *luciferase* gene: 5′-CTTCGAAATGTCCGTTCGGTT-3′.

### Phosphopeptide enrichment using IMAC

For packing IMAC column, a 10 cm microcolumn (500 μm id PEEK column, Upchurch Scientific/Rheodyne) that has been packed with Ni-NTA resin (Qiagen) was enclosed in a stainless-steel column-end fitted with a 0.5 μm frit disk at one end. Phosphopeptide purification was performed using an autosampler and an HP1100 solvent delivery system (Hewlett-Packard). The flow rate was set at 13 μl min^−1^ and the loading/condition buffer was 6% (v/v) AA with pH adjusted to 3.0. Ni^2+^ ions on the resin were removed by an amount of 100 μl of 50 mM EDTA in 1 M NaCl followed by equilibration with loading buffer for 15 min. To equip the IMAC column with Fe^3+^, 100 μl of 0.2 M FeCl_3_ was loaded into the column for another 15 min before sample loading. Peptide samples were resolved in loading buffer and loaded into the Fe^3+^-equipped IMAC column for 20 min. A total of 100 μl of 25% (v/v) acetonitrile was used to wash the unbound peptides away for 15 min. The bound peptides were eluted with 100 μl of 200 mM NH_4_H_2_PO_4_ and dried by vacuum centrifugation for further use.

### Liquid chromatography–MS/MS analysis

Liquid chromatography–MS/MS analysis was performed on an LTQ-Orbitrap XL mass spectrometer (Thermo Fisher Scientific, Bremen, Germany) for phosphoproteome, which equipped with a nanospray interface (Proxeon, Odense, Denmark). Peptides were separated on a nanoAcquity system (Waters, Milford, MA), which was connected to mass spectrometry. Peptide mixtures were loaded onto a 75 μm ID, 25 cm length C18 BEH column (Waters, Milford, MA) packed with 1.7 μm particles with a pore of 130 Å. A segmented gradient in 90 min from 1 to 35% solvent B (acetonitrile with 0.1% formic acid) at a flow rate of 300 nl min^−1^ and a column temperature of 35 °C were used. Solvent A was 0.1% formic acid in water. Survey full-scan MS spectra were acquired in the orbitrap (*m*/*z* 350–1,600) with the resolution set to 60,000 at *m*/*z* 400 and automatic gain control (AGC) target at 10^6^. The mass spectrometer was operated in the data-dependent mode. The 10 most intense ions were sequentially isolated for CID MS/MS fragmentation and detected in the linear ion trap (AGC target at 7,000) with previously selected ions dynamically excluded for 90 s. Ions with singly and unrecognized charge state were excluded. To improve the fragmentation spectra of the phosphopeptides, ‘multistage activation' at 97.97, 48.99 and 32.66 Thomson (Th) relative to the precursor ion was enabled in all MS/MS events. All the measurements in the Orbitrap were performed with the lock mass option for internal calibration.

### Phosphopeptide identification and label-free quantification

Raw MS/MS data were converted into peak lists (MGF file) using Raw2MSM (ref. 41) with default parameters. The resulting peak lists were searched against the IPI_MOUSE_3.87 database (version 3.87, 68161 entries) via an in-house Mascot search engine (Matrix Science Ltd., UK; version 2.2.1). The search parameters were set as follows: peptide mass tolerance, 10 p.p.m. MS/MS ion mass tolerance, 0.6 Da; enzyme set as trypsin and allowance of up to two missed cleavages; variable modifications included oxidation on methionine and phosphorylation on serine, threonine and tyrosine residues; peptide charge, 2+ and 3+. Phosphopeptide identification results were accepted only if the Mascot scores pass statistically confidence (*P*<0.05) and the identified sequence ranked as the top match. The search results in MASCOT were exported in eXtensive Markup Language data (.XML) format. The raw data were converted into files of mzXML format. Peptide identification results from each liquid chromatography–MS/MS run and the corresponding XML files were used for quantitative analysis by IDEAL-Q as described previously^16^,^17^.

### Statistics

Statistical significance was analysed by two-tailed unpaired *t*-tests in which the variances of the groups are similar. A value of *P*<0.05 was considered significant. All the data shown in this study are the mean±s.e.m. from at least three biological replicates.

### Data availability

Accession codes: The raw data sets have been deposited in the ProteomeXchange Consortium (http://proteomecentral.proteomexchange.org) via the PRIDE^42^ partner repository with the data set identifier PXD003788. The additional data that support the findings of this study are available from the corresponding author on request.

## Additional information

**How to cite this article:** Wu, J.-L. *et al*. Temporal regulation of Lsp1 *O*-GlcNAcylation and phosphorylation during apoptosis of activated B cells. *Nat. Commun.* 7:12526 doi: 10.1038/ncomms12526 (2016).

## Supplementary Material

Supplementary Information Supplementary Figures 1-10

Supplementary Data 1 Summary of quantitative analysis of phosphoproteins in mouse splenic B cells with or without anti-IgM or TG treatment

## Figures and Tables

**Table 1:** Summary of phosphoproteome changes in the number of phosphorylation sites and proteins.

	**No. of sites**	**%**	**No. of proteins**	**No. of peptides**		**No. of sites**	**%**	**No. of proteins**	**No. of peptides**
**Ratio: B/A<0.5**					**Ratio: B/A>2**				
pS	460	86.96	327		pS	536	84.68	361	
pT	67	12.67	62		pT	80	12.64	68	
pY	2	0.38	2		pY	17	2.69	15	
Total	529		349	553	Total	633		376	672
**Ratio: C/B<0.5**					**Ratio: C/B>2**				
pS	89	92.71	67		pS	201	92.63	154	
pT	5	5.21	5		pT	16	7.37	15	
pY	2	2.08	2		pY	0	0.00	0	
Total	96		70	101	Total	217		162	225

Mouse splenic B cells were treated with either anti-IgM (condition B) or TG before the anti-IgM treatment (condition C); untreated B cells were used as the control (condition A).
