# Supplementary material for: Temporal regulation of Lsp1 O-GlcNAcylation and phosphorylation during apoptosis of activated B cells
Source: Nat Commun. 2016 Aug 24;7:12526. doi: 10.1038/ncomms12526 (PMC4999498; doi:10.1038/ncomms12526)
Supplement: Supplementary Information — Supplementary Figures 1-10 [file ncomms12526-s1.pdf]

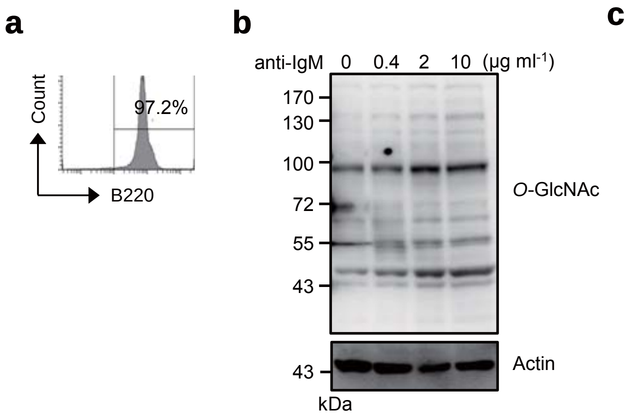

Supplementary Figure 1. Accumulation of *O*-GlcNAcylated Proteins in Anti-IgM Stimulated Mouse Splenic B Cells

a. Flow cytometric analysis showing the purity of isolated mouse splenic B cells.

b. IB showing the levels of *O*-GlcNAcylated proteins in splenic B cells after treatment with various doses of anti-IgM (0, 0.4, 2 and 10 μg ml^{-1}) at 24 h.

c. IB showing the levels of OGT in mouse primary splenic B cells after anti-IgM (10 μg ml^{-1}) stimulation at various time points.

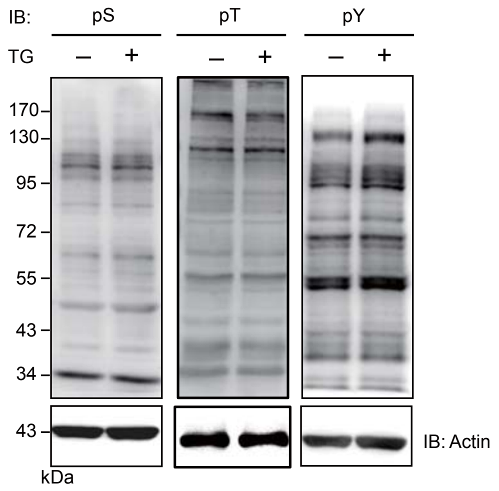

Supplementary Figure 2. Effects of Elevated *O*-GlcNAcylation on Protein Phosphorylation

IB showing the levels of various global phosphorylated proteins in primary mouse splenic B cells that were pre-treated with or without 1.0 μM TG for 8 h and then stimulated with anti-mouse IgM (10 μg ml^{-1}) for 30 min. The antibodies against global Serine (S), Threonine (T) and Tyrosine (Y) phosphorylation levels are used.

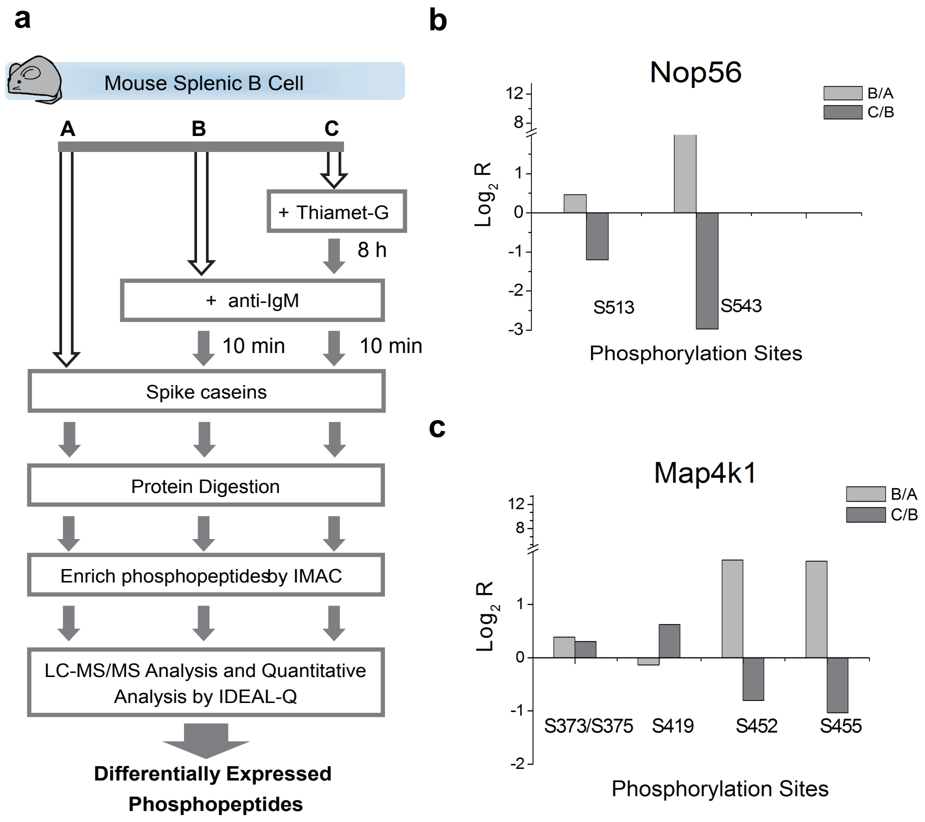

Supplementary Figure 3. Quantitative Proteomics Approach Used to Delineate Global Interplay between Phosphorylation and *O*-GlcNAcylation

a. Schematic flowchart of label-free phosphoproteomics-based identification of protein phosphorylation events. A total of 800 μg of proteins isolated from mouse splenic B cells treated with anti-IgM (10 μg ml^{-1}) for 0 or 10 min or with additional pretreatment of TG were digested with trypsin. Before protein digestion, a standard phosophoprotein, β-casein, was spiked into each sample to control the variation from sample preparation and instrument. Phosphopeptides were purified with iron-NTA IMAC and analyzed by liquid chromatography coupled tandem mass spectrometry. Quantification was achieved using IDEAL-Q software.

b. Phosphorylation levels of 513 and S543 on Nop56 were not changed and increased after anti-IgM (10 μg ml^{-1}) treatment, respectively, but both decreased due to pretreatment of TG.

c. The levels of phosphorylation of S373/S375 on Map4k1 were not affected by the treatment with TG while the other two phosphorylation sites, S452 and S455, were decreased due to the enhanced *O*-GlcNAcylation. However, S419 showed the reverse effect.

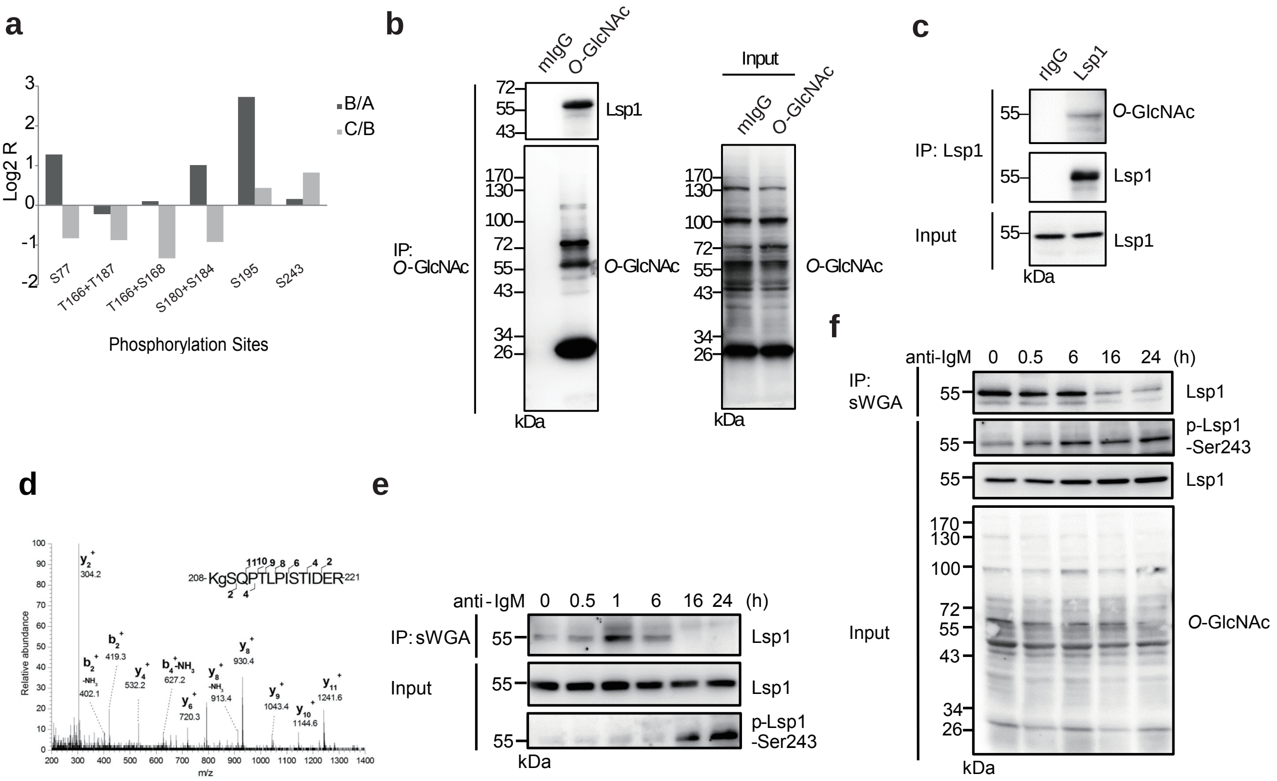

Supplementary Figure 4. Confirmation of *O*-GlcNAcylation and Modification Sites on Lsp1

a. Mapped phosphorylation sites on Lsp1 that are sensitive to TG treatment (sites that have both B/A and C/B ratios were depicted).

b and c. Cell lysates were prepared from BCL-1 cells and immunoprecipitated with anti-*O*-GlcNAc (b) or anti-Lsp1 (c). Immunoprecipitates and input lysates were analyzed by IB using the indicated antibodies.

d. Mapping of *O*-GlcNAcylation site on Lsp1 by using CID fragmentation during MS/MS analysis. The b_2^+ ion (419.3 Da) suggested that S209 carried a GlcNAc moiety.

e. IB showing the levels of Lsp1, S243 phosphorylated Lsp1, and Lsp1 *O*-GlcNAcylation by sWGA pull-down in anti-IgM (10 μg ml^{-1}) stimulated mouse splenic B cells at indicated time points.

f. IB showing the levels of Lsp1, S243 phosphorylated Lsp1, and Lsp1 *O*-GlcNAcylation by sWGA pull down in TG treated and anti-IgM (10 μg ml^{-1}) stimulated mouse splenic B cells at indicated time points.

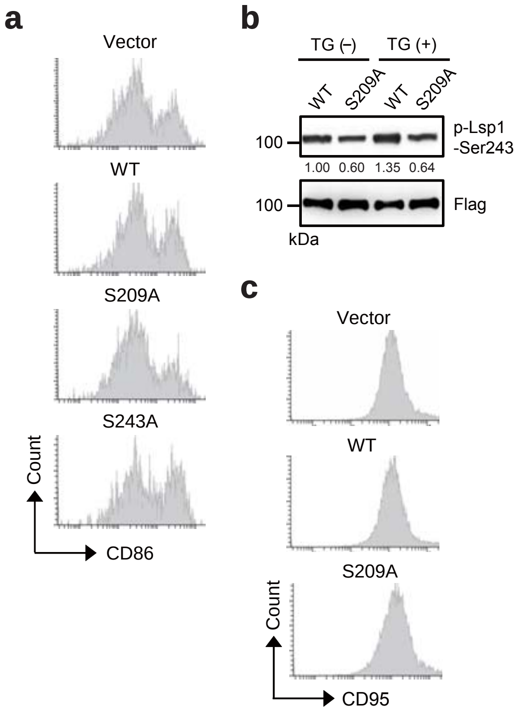

Supplementary Figure 5. B Cell Activation and CD95 Expression Are Not Affected by Lsp1 and its *O*-GlcNAc Site Mutant

a. Lsp1 does not affect B cell activation. Primary mouse splenic B cells were transduced with retroviral vector, WT or S209A Lsp1 and were stimulated with anti-mouse IgM (10 μg ml^{-1}) for 48 h. Flow cytometric analysis was used to detect the levels of activation marker, CD86, on transduced YFP^+ cells.

b. IB showing the levels of S243 phosphorylated Lsp1 in sorted $EGFP^+$ Ramos B cells expressing Flag-EGFP-tagged WT or S209A Lsp1 in the absence and presence of TG treatment.

c. S209A mutant does not affect surface CD95 expression. Primary mouse splenic B cells were transduced with retroviral vector, WT or S209A Lsp1 and were stimulated by anti-mouse IgM (10 μg ml^{-1}) for 48 h. The level of CD95 on transduced YFP^+ cells was checked by flow cytometric analysis.

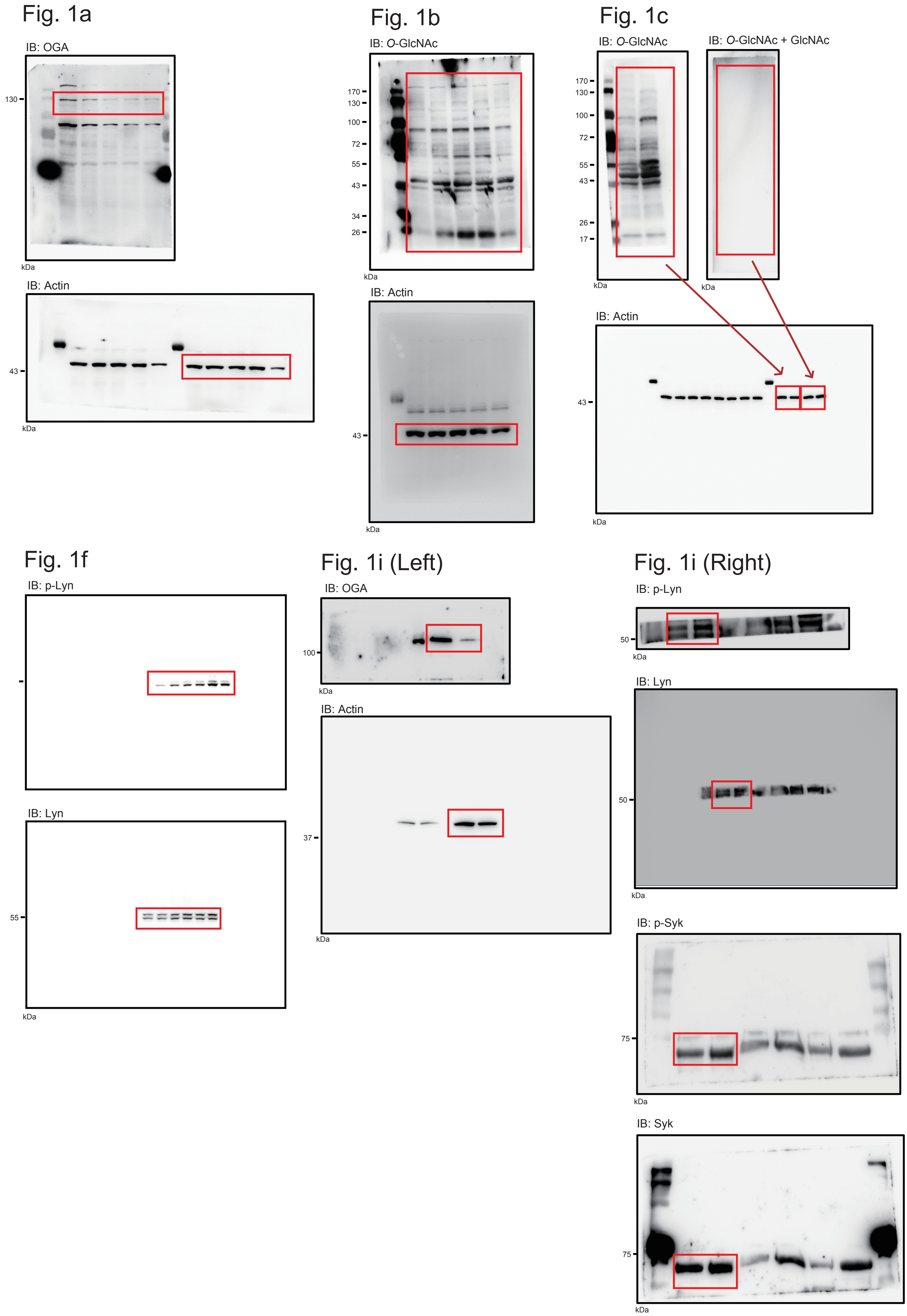

Supplementary Figure 6. Uncropped scanned images of immunoblots from Figure 1

Fig. 3c

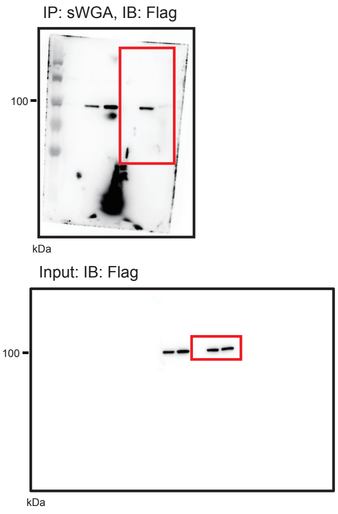

Fig. 3d

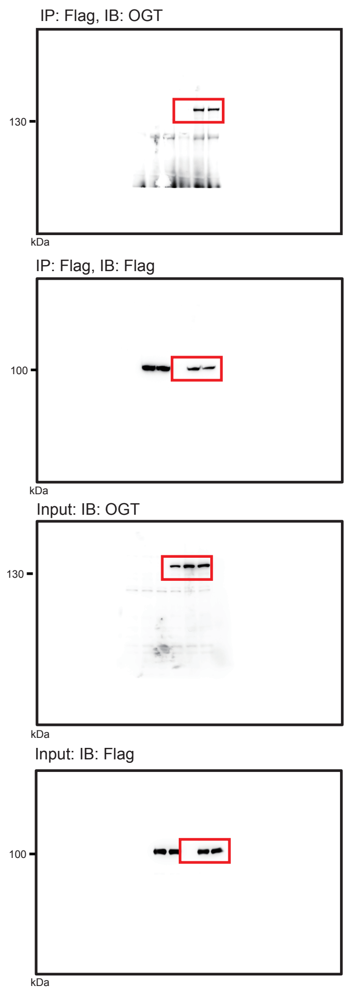

Fig. 3e

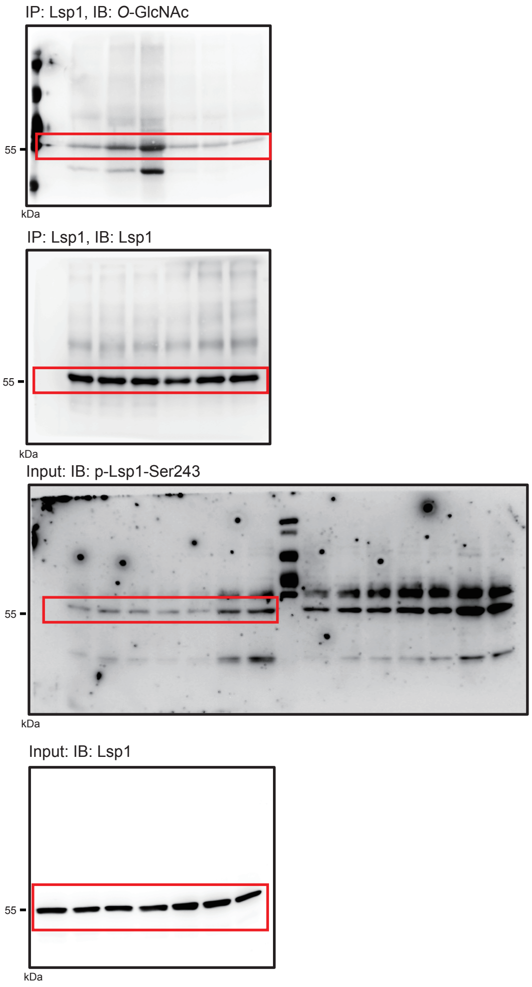

Fig. 3f

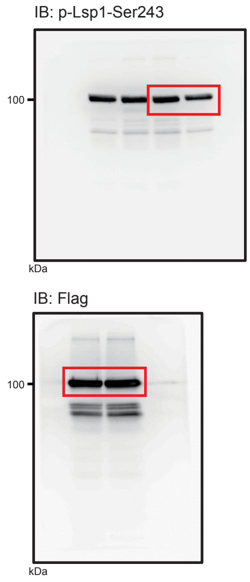

Fig. 3g

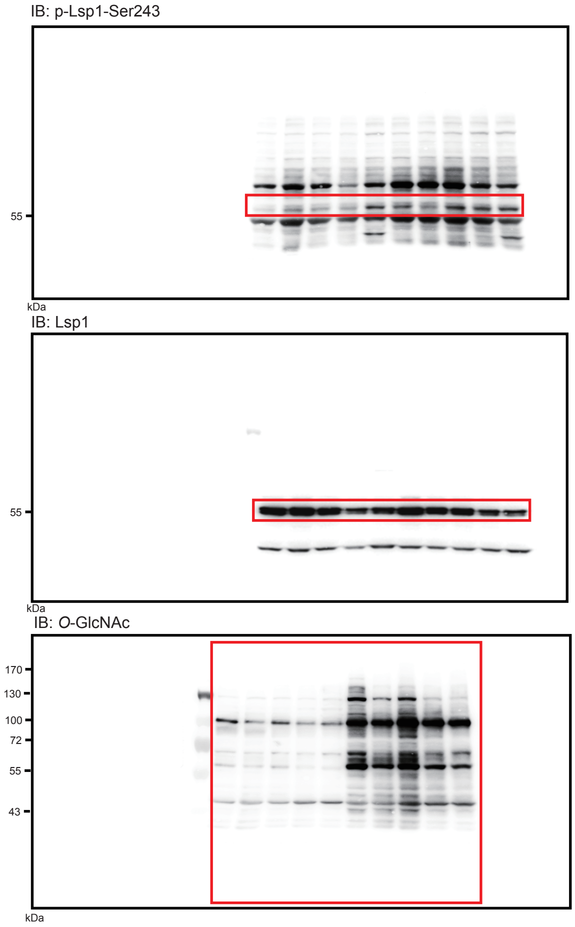

Supplementary Figure 7. Uncropped scanned images of immunoblots from Figure 3

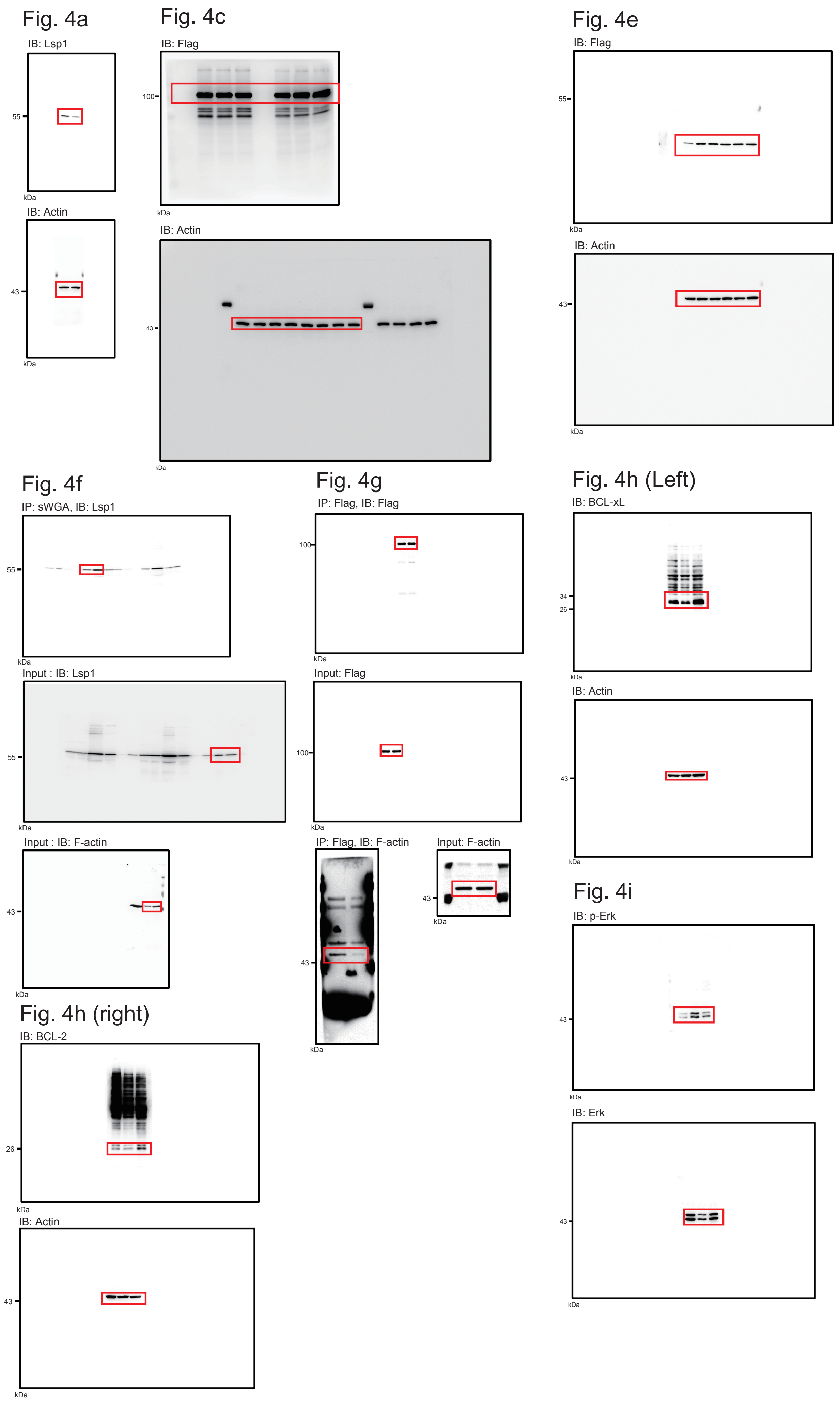

Supplementary Figure 8. Uncropped scanned images of immunoblots from Figure 4

Fig. 5a

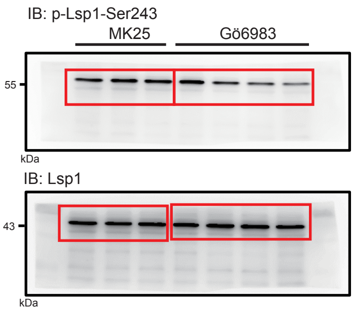

Fig. 5c

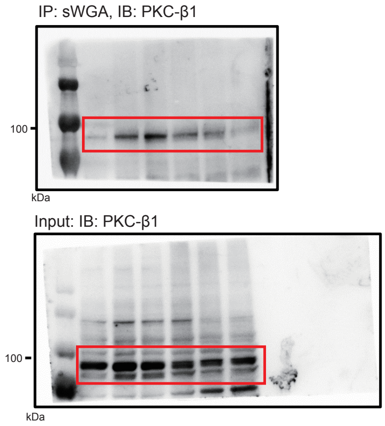

Fig. 5d

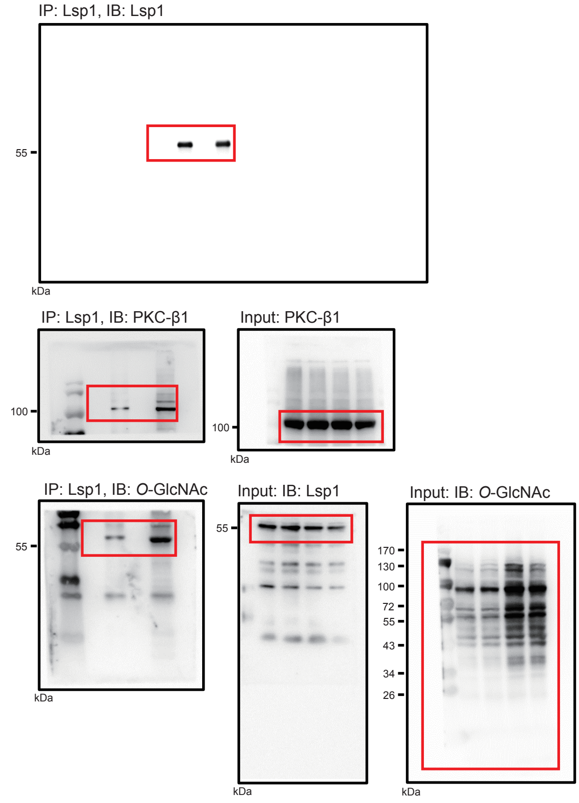

Fig. 5e

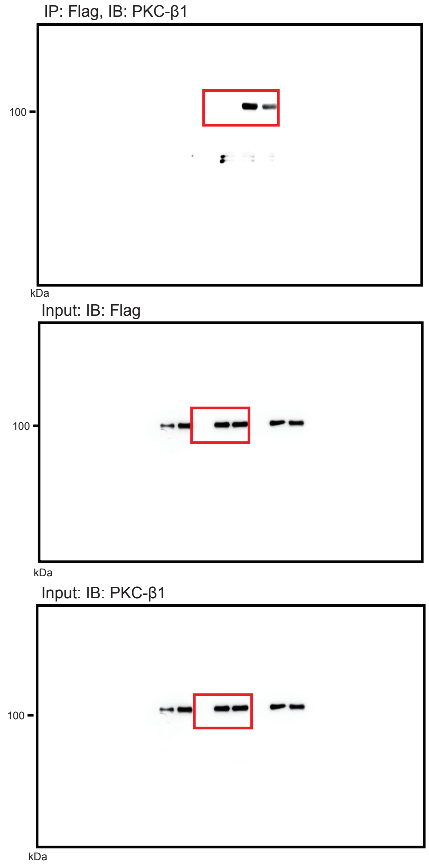

Supplementary Figure 9. Uncropped scanned images of immunoblots from Figure 5

Sup. Fig. 1b

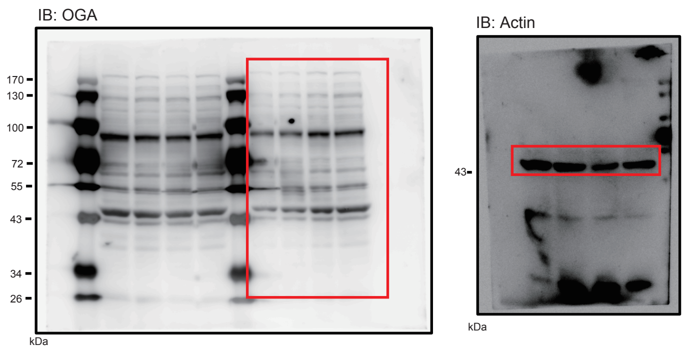

Sup. Fig. 1c

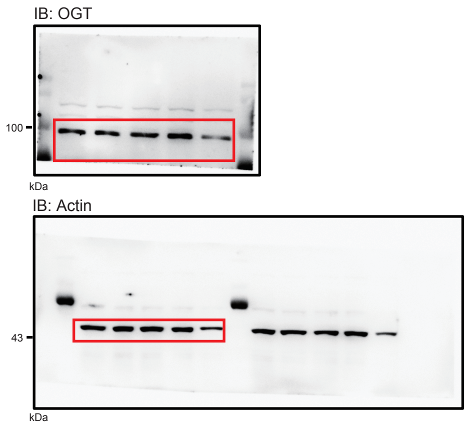

Sup. Fig. 2 (Left)

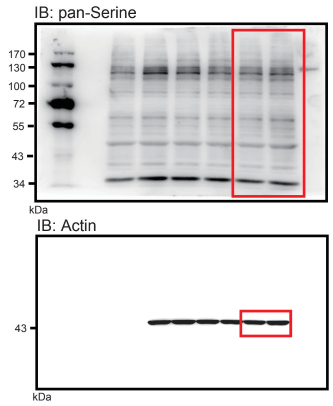

Sup. Fig. 2 (Middle)

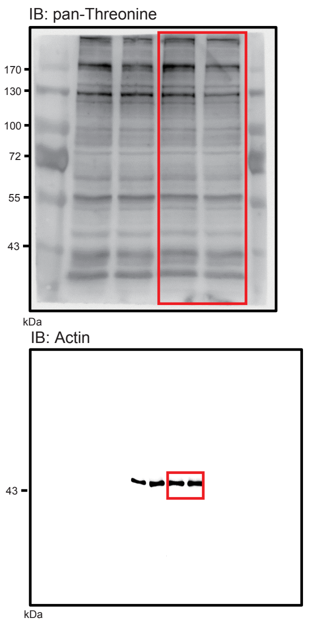

Sup. Fig. 2 (Right)

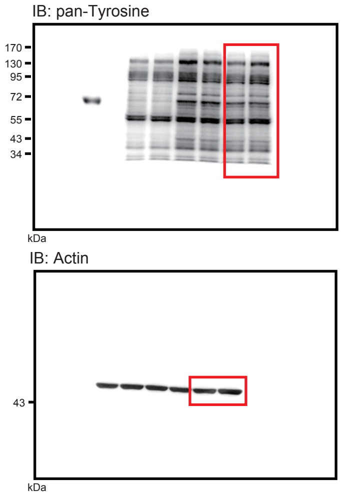

Sup. Fig. 4b

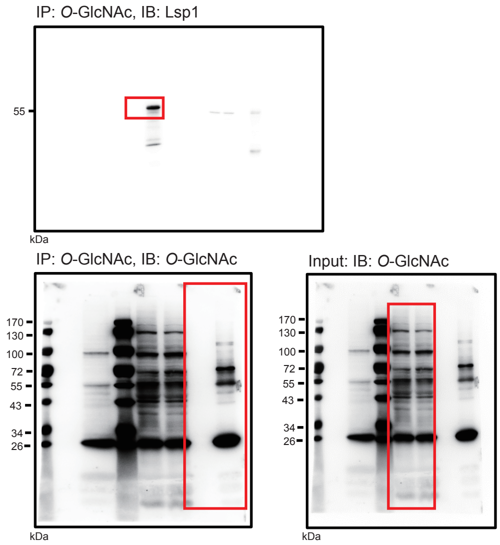

Sup. Fig. 4c

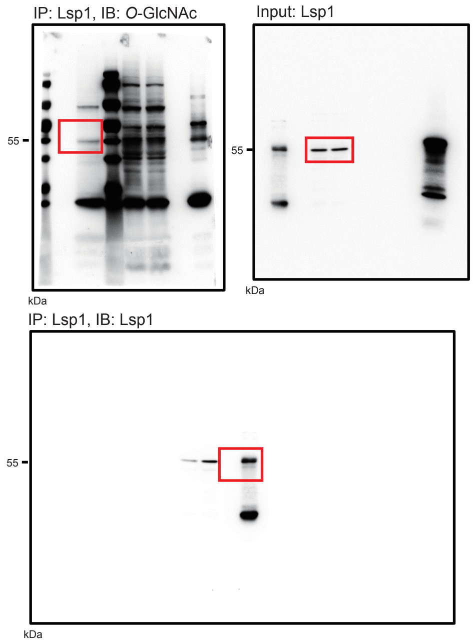

Sup. Fig. 4f

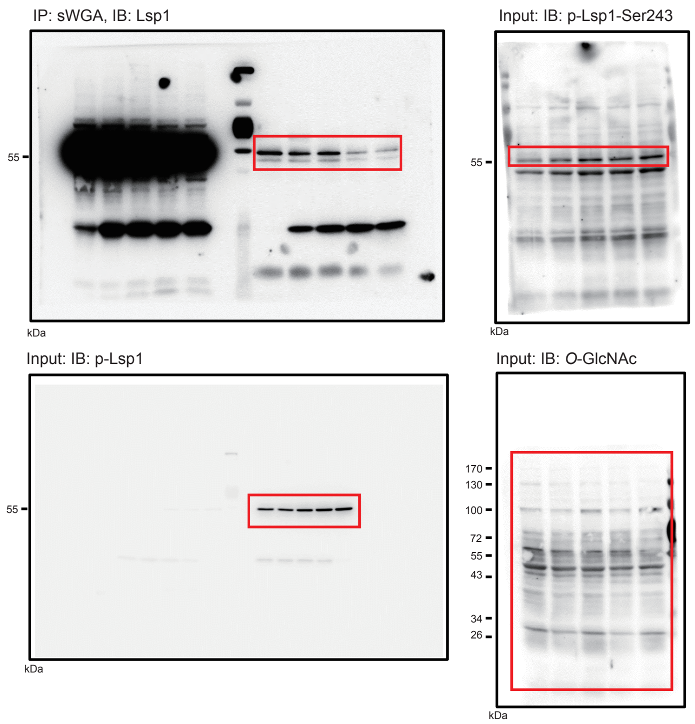

Sup. Fig. 5b

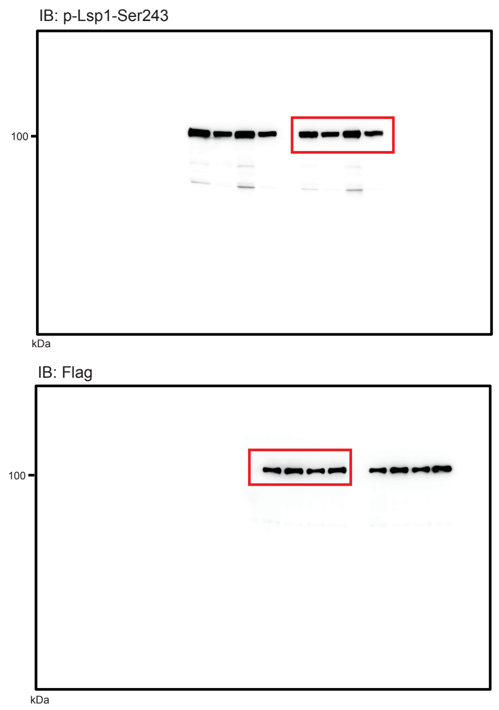

Supplementary Figure 10. Uncropped scanned images of immunoblots from Supplementary Figures
